# Calciphylaxis Secondary to Vitamin D Supplementation

**DOI:** 10.7759/cureus.44778

**Published:** 2023-09-06

**Authors:** Joana Costa e Silva, José N Ramos

**Affiliations:** 1 Plastic Reconstructive and Maxilofacial Surgery, Centro Hospitalar de Lisboa Ocidental, Lisbon, PRT

**Keywords:** end-stage kidney disease, non-uremic calciphylaxis, calcific uremic arteriolopathy, vitamin d, calciphylaxis

## Abstract

Calciphylaxis is a rare cutaneous disease consisting of purpuric and necrotic lesions, and it affects mostly patients with renal failure. More rarely, it can be observed in patients with preserved renal function, acquiring the name of non-uremic calciphylaxis (NUC). Although its pathophysiology is under uncertainty, many etiological factors have been implicated. This report describes a patient who developed NUC, possibly triggered by vitamin D supplementation.

## Introduction

Calciphylaxis, also known as calcific uremic arteriolopathy (CUA), is a rare cutaneous condition that occurs most frequently in patients with end-stage kidney disease (ESKD) [1] and presents as purpuric skin lesions that evolve to necrotic ulcers. Although the mechanisms of the disease are not fully understood, it is assumed that hyperparathyroidism, hyperphosphatemia, hypercalcemia, and vitamin D supplementation secondary to uremia contribute to CUA [1-5]. Calciphylaxis has also been reported in patients without ESKD, being denominated non-uremic calciphylaxis (NUC), with a high mortality rate around 50%, mainly due to sepsis [3]. This rarer form of the disease is also uncertain regarding to its pathophysiology, but several risk factors have been suggested, such as malignancies, primary hyperparathyroidism, and chronic liver disease. Its diagnosis requires a high level of suspicion, based on the recognition of characteristic clinical features in patients with known risk factors and on a skin biopsy [3]. Although rare, this condition is experiencing an increased reporting, possibly due to greater awareness.

Herein, we describe a case of NUC on a female patient possibly triggered by vitamin D supplementation.

## Case presentation

A 71-year-old female patient was referred to a plastic surgery consultation for a single painful necrotic skin lesion on the right leg. She had a past medical history of obesity and well-controled diabetes mellitus type II over the past 20 years. Her chronic medication included daily metformin 1000 mg and also vitamin D3 20000 IU for the past six months, prescribed by her family doctor to prevent osteoporosis. Initially, the physical exam showed a 2 x 2 cm painful necrotic skin ulcer on the right leg, with stellate borders and a black eschar, surrounded by purpuric skin. The leg presented warm and edematous and with a poorly demarcated erythema. No sepsis signs were registered. The patient was hospitalized for analgesic and antibiotic therapy and wound care.

Laboratory studies were consistent with normal kidney function. Parathyroid hormone, 25-hydroxy-vitamin D, calcium, and phosphate levels were within normal range. Screening for vasculitis, thrombophilia, and malignancies was also negative. Skin biopsy was consistent with NUC. After investigation of possible risk factors, vitamin D supplementation was immediately suspended.

**Figure 1:**
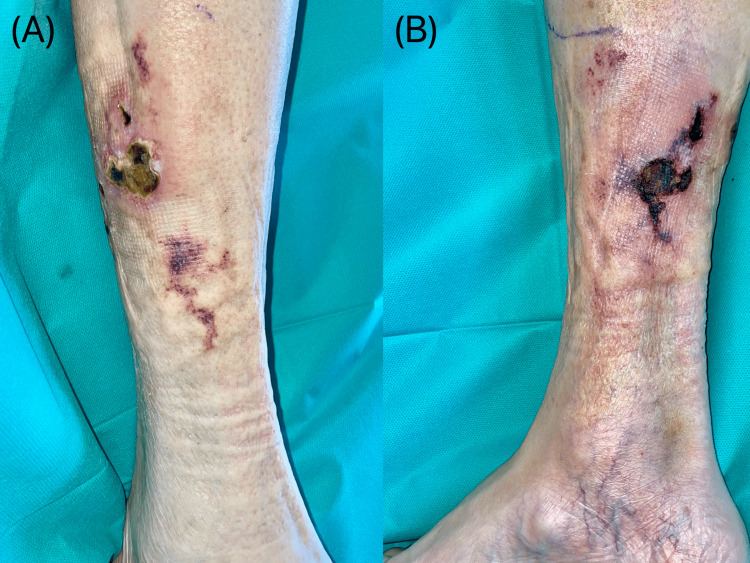
Necrotic skin ulcer on the right leg, with stellate borders and a black eschar, surrounded by purpuric skin. (A) Lateral side of the right leg. (B) Medial side of the right leg.

Following a two-week period characterized by new lesion development (Fig. 1), it finally began to stabilize. As the lesions continued to be limited to the leg with no associated systemic signs, it was decided to continue daily wound care with silver sulfadiazine cream and to decline invasive surgical interventions.

**Figure 2:**
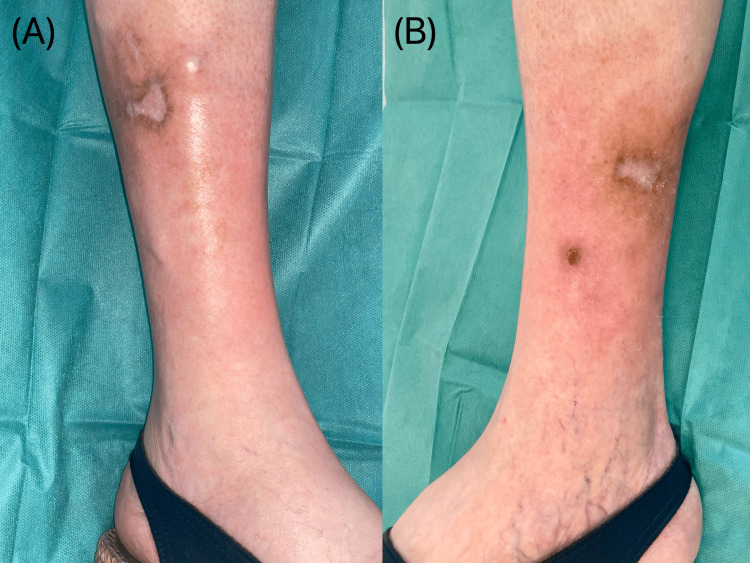
Skin lesions healing after six months of wound care. (A) Lateral side of the right leg. (B) Medial side of the right leg.

After one month of hospitalization, the patient was discharged with the ulcers in a good healing phase. After six months, the ulcers were completely healed (Fig. 2).

## Discussion

Calciphylaxis, first described by Selye et al. [6], is a syndrome characterized by purpuric skin lesions that progress to painful necrotic ulcers, mainly in the lower limbs. Pathological mechanisms are not well understood but involve vascular calcification and fibrosis due to elevated calcium phosphorus product [4]. Skin biopsy usually shows medial arteriolar calcification and thrombotic occlusion of cutaneous vessels, which leads to ischemic necrosis of the subcutis, dermis, or epidermis with extravascular deposition [1]. It seems that the mechanisms involved in vascular calcification are the same ones involved in bone metabolism, and all appear to have a nuclear factor k-B (NFkB) activation in common. NFkB activity comprises the receptor activator of NFkB (RANK) and its ligand (RANKL) and also osteoprotegerin (OPG), which is an RANKL antagonist. The RANK-NFkB pathway is essential for normal bone development, osteoclast differentiation, and bone mineral resorption; increased activity causes osseous mineral loss and vascular mineral deposition. Expression of RANKL or OPG may be modified by numerous cytokines, medications, hormones, and other agents. In fact, parathyroid hormone, corticosteroids, and liver disease are known to increase the expression of RANKL and decrease the expression of OPG, thus activating the NFκB pathway. Other vascular calcification inhibitors, such as fetuin-A and matrix Gla protein, may also play a role in CUA [7-8].

In the non-uremic type, the pathophysiology has been less investigated, but it is fair to say that its pathogenesis likely shares some of the same mechanisms behind the CUA. It has been reported in patients with different comorbidities, such as primary hyperparathyroidism, connective tissue diseases, alcoholic liver disease, Crohn's disease, and malignancies. Other risk factors include female sex, diabetes, obesity, warfarin therapy, corticosteroid medication, and vitamin D supplementation [3]. The treatment of this often fatal disease involve withdraw of any possible trigger, analgesia and antibiotic optimization, and careful surgical or chemical debridement of the lesions [2].

In the presented case, after careful investigation, the only risk factor found was vitamin D supplementation, in addition to the known risk factors, such as female gender, obesity, and diabetes.

Vitamin D was studied in Selye et al.'s model as a pro-calcification agent [6]. However, its role in vascular calcification is not that clear. While it may be responsible for inducing hypercalcemia and hyperphosphatemia, it may also act as a vascular protector when taken as a supplement. Several studies have demonstrated this vascular protection with both vitamin D deficiency and supplementation through various mechanisms, including modulation of vascular regeneration, alteration in the RANLK/OPG activity, and increase in calcification inhibitors, such as fetuin-A [7,9].

In fact, vitamin D supplementation has been considered a precipitating factor on the uremic-type calciphylaxis, specifically in patients with severely dysregulated calcium-phosphorus metabolism, usually on dialysis, or in combination with calcium therapy [4]. In the non-uremic type, vitamin D deficiency seems to be the cause [3], with few reports claiming otherwise [10].

In this specific case, in a patient with normal kidney function, it is reasonable to correlate NUC with vitamin D oversupplemention. The tolerable upper limit, defined as the highest level of daily nutrient intake that is likely to pose no risk of adverse health effects, is 4,000 IU per day for adults, although short-term ingestion up to 10,000 IU does not show toxicity [11]. This patient has taken about 20,000 IU daily for the past six months, which appears to be a high concentration for a patient with likely previous normal or near-normal serum calcium levels. Although 25-hydroxy-vitamin D, calcium, and phosphate levels were within normal range, this could be due to those mineral consumption, or vitamin D could have caused the disease by a mechanism unrelated to calcium-phosphorus metabolism.

Although difficult to prove, the fact that the disease started to improve two weeks after stopping vitamin D supports the hypothesis of it as a risk factor. Combined with the fact that the screening for the main causes of NUC was negative, one should consider vitamin D as a trigger, alongside female sex, obesity, and diabetes.

The management of calciphylaxis includes removal of any possible trigger, analgesia and antibiotic optimization, and careful wound care. Chemical debridement was preferred, as the lesions were composed of stable, dry, non-infected eschars. Surgical debridement should be chosen when the lesions become infected, considering that the primary cause of death is sepsis [2]. Sodium thiosulfate has been used off-label with some successful outcomes, mostly on uremic calciphylaxis, but a recent meta-analysis found no improvement in skin lesions or survival [12]. To a large extent, more efficient therapeutic measures and evidence-based recommendations are needed.

## Conclusions

Calciphylaxis remains a challenging condition, with many etiopathological contributors and an often fatal outcome. In this particular case, vitamin D may have triggered its development. Although reports on the association between vitamin D supplementation and NUC are few, the numbers may be higher due to the increased vitamin D supplementation in recent years. Therefore, it is important to consider vitamin D supplementation as a risk factor in the evaluation of the disease.
